# Novel chemoimmunotherapeutic strategy for hepatocellular carcinoma based on a genome-wide association study

**DOI:** 10.1038/srep38407

**Published:** 2016-12-02

**Authors:** Kaku Goto, Dorcas A. Annan, Tomoko Morita, Wenwen Li, Ryosuke Muroyama, Yasuo Matsubara, Sayaka Ito, Ryo Nakagawa, Yasushi Tanoue, Masahisa Jinushi, Naoya Kato

**Affiliations:** 1 The Advanced Clinical Research Center, The Institute of Medical Science, The University of Tokyo, Tokyo 108-8639, Japan; 2 Japan Society for the Promotion of Science, Tokyo 102-8472, Japan; 3 Institute for Genetic Medicine, Hokkaido University, Hokkaido 060-0815, Japan; 4 Institute for Advanced Medical Research, Keio University Graduate School of Medicine, Tokyo 160-8582, Japan

## Abstract

Pharmacotherapeutic options are limited for hepatocellular carcinoma (HCC). Recently, we identified the anti-tumor ligand MHC class I polypeptide-related sequence A (*MICA*) gene as a susceptibility gene for hepatitis C virus-induced HCC in a genome-wide association study (GWAS). To prove the concept of HCC immunotherapy based on the results of a GWAS, in the present study, we searched for drugs that could restore MICA expression. A screen of the FDA-approved drug library identified the anti-cancer agent vorinostat as the strongest hit, suggesting histone deacetylase inhibitors (HDACis) as potent candidates. Indeed, the HDACi-induced expression of MICA specific to HCC cells enhanced natural killer (NK) cell-mediated cytotoxicity in co-culture, which was further reinforced by treatment with an inhibitor of MICA sheddase. Similarly augmented anti-tumor activity of NK cells via NK group 2D was observed *in vivo*. Metabolomics analysis revealed HDACi-mediated alterations in energy supply and stresses for MICA induction and HCC inhibition, providing a mechanism for the chemoimmunotherapeutic actions. These data are indicative of promising strategies for selective HCC innate immunotherapy.

Hepatocellular carcinoma (HCC) remains a leading cause of cancer-related mortality, claiming the lives of 700,000 individuals annually worldwide^1^. In the vast majority of cases, the etiology of HCC involves carcinogenic viruses such as hepatitis B virus (HBV) and hepatitis C virus (HCV) in the vast majority of cases, and the disease is becoming increasingly more controllable; nevertheless, HCC is a heterogeneous disease^2^ with a highly intricate mechanism of development^3^, and thus many questions related to its etiology and pathogenesis remain unanswered. The current pharmacotherapeutic options for tumor surveillance and elimination are limited owing to the absence of specific critical targets and the high frequency of the development of chemoresistance^4^. We recently performed a genome-wide association study (GWAS) and identified the immunoactivating anti-tumor ligand MHC class I polypeptide-related sequence A (*MICA*) gene as susceptibility gene for HCV-induced HCC^5^. Furthermore, lower levels of *MICA* expression were associated with a higher risk of HCC development in patients, and shedding of MICA is known to interdict its action^6^, indicating that the hypofunction of anti-cancer immunity is a suitable target for pharmacotherapy via manipulating MICA expression.

The unprecedented efficacy of cancer immunotherapy is increasingly being recognized^7^. The aim of the present study was to prove the concept of HCC immunity restoration through editing of target cells, i.e., the pharmacological induction of MICA expression. Toward this end, we established a functional luciferase reporter cell clone of *MICA* promoter activity. Subsequently, we screened the FDA-approved drug library, and identified the anti-cancer agent vorinostat (VOR), a histone deacetylase (HDAC) inhibitor (HDACi), as the overwhelmingly strongest hit. We then tested the induction of MICA specifically in HCC cells by HDACis including VOR in combination with shedding inhibition and accompanied enhancement of natural killer (NK) cell-mediated cytotoxicity through MICA-NK group 2D (NKG2D) signaling in co-culture and *in vivo*. Furthermore, metabolomics analysis specifically uncovered the altered energy supply and stress pathways responsible for MICA induction and HCC cell inhibition, giving a physiological explanation of the mechanism underlying the chemoimmunotherapeutic efficacy of HDACi. These results provide not only a proof of concept but also suggest promising strategies for selective HCC innate immunotherapy to overcome the intricacies of carcinogenesis, as the first example of GWAS-based medicine.

## Results

### Generation of a reporter cell system for *MICA* promoter activity

**Figure 1:**
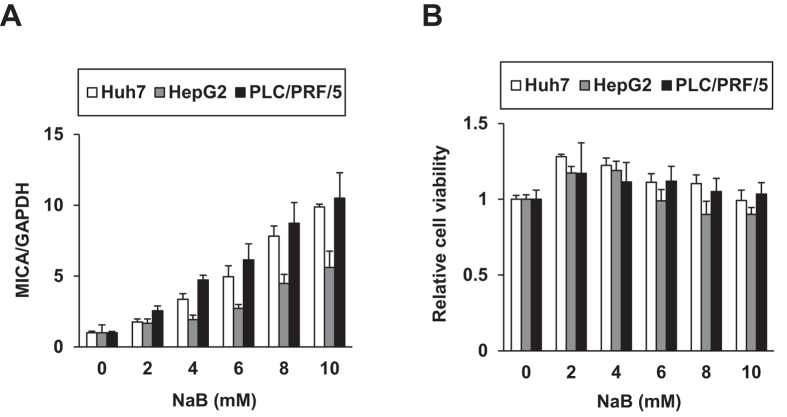
NaB upregulated *MICA* expression in hepatoma cells. After treatment with NaB for 48 h, relative *MICA* mRNA levels were quantified by qRT-PCR with normalization to *GAPDH* (**A**), and cell viabilities were determined by a tetrazolium salt assay (**B**) in three hepatoma cell lines: Huh7, HepG2, and PLC/PRF/5.

**Figure 2:**
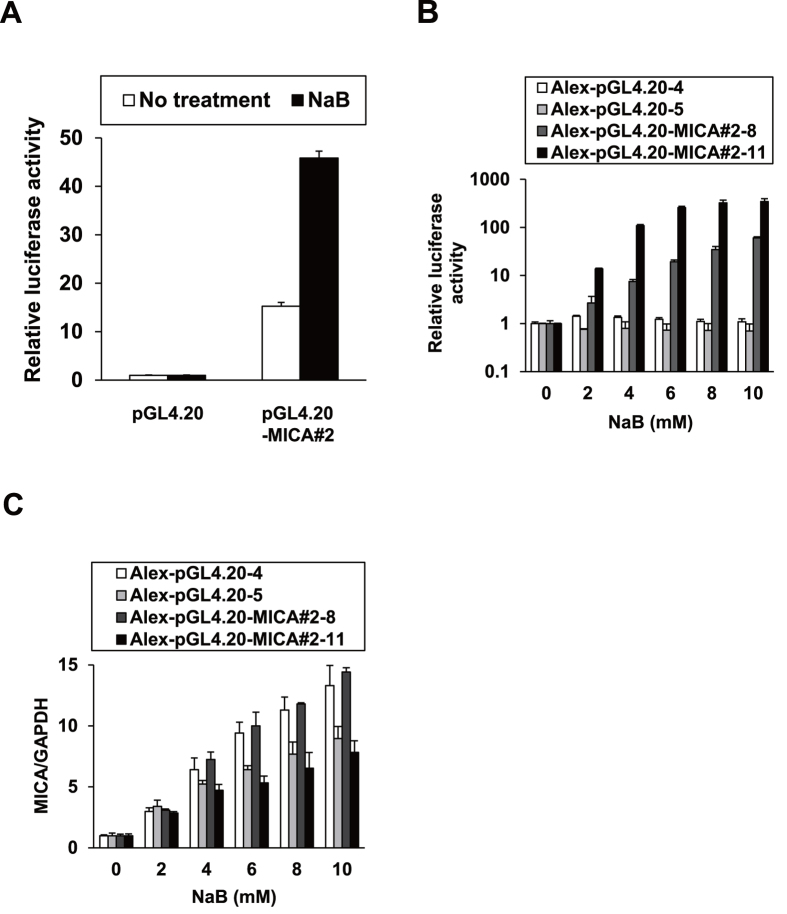
NaB enhanced *MICA* promoter activity in the reporter system. (**A**) PLC/PRF/5 cells were transfected with either pGL4.20 or pGL4.20-MICA#2 with pRL-TK for 24 h followed by NaB treatment for 24 h, and then the cells were lysed for a dual luciferase assay. (**B**) The control cell clones Alex-pGL4.20-4 and -5 and the reporter cell clones Alex-pGL4.20-MICA#2-8 and -11 were treated with NaB for 48 h, and then lysed for a luciferase assay. Firefly luciferase activity was normalized to cell viability determined by a tetrazolium salt assay immediately before cell lysis, yielding the relative luciferase activity. (**C**) Total RNA was extracted from the cell clones treated as described in (**B**), and *MICA* mRNA levels were quantified by qRT-PCR with normalization to *GAPDH*.

We first ascertained the pharmacological upmodulation of MICA expression in hepatoma cells. Huh7, HepG2, and PLC/PRF/5 (Alexander) cells were treated with sodium butyrate (NaB), a reported MICA expression inducer^8^. Indeed, NaB enhanced *MICA* mRNA expression levels (Fig. 1A) without causing cytotoxicity (Fig. 1B). We then constructed a reporter system for *MICA* promoter activity; the approximately 1-kb promoter region covering reported sequences^9^,^10^ was cloned in the pGL4.20 luciferase reporter vector, producing pGL4.20-MICA#2. In PLC/PRF/5 cells, the luciferase activity of the reporter was upregulated by NaB (Fig. 2A). Subsequently, stable PLC/PRF/5 cell clones with the vectors were established by puromycin selection, producing the control cell clones Alex-pGL4.20-4 and -5 and the clones harboring the *MICA* promoter reporter, Alex-pGL4.20-MICA#2-8 and -11. Luciferase activity increased in a dose-dependent manner in response to NaB treatment, specifically in the reporter cell clones (Fig. 2B), with concurrent elevations in *MICA* mRNA levels (Fig. 2C). These results indicated that the reporter system was successfully generated to reflect *MICA* promoter activity.

### Screen of the FDA-approved drug library

**Figure 3:**
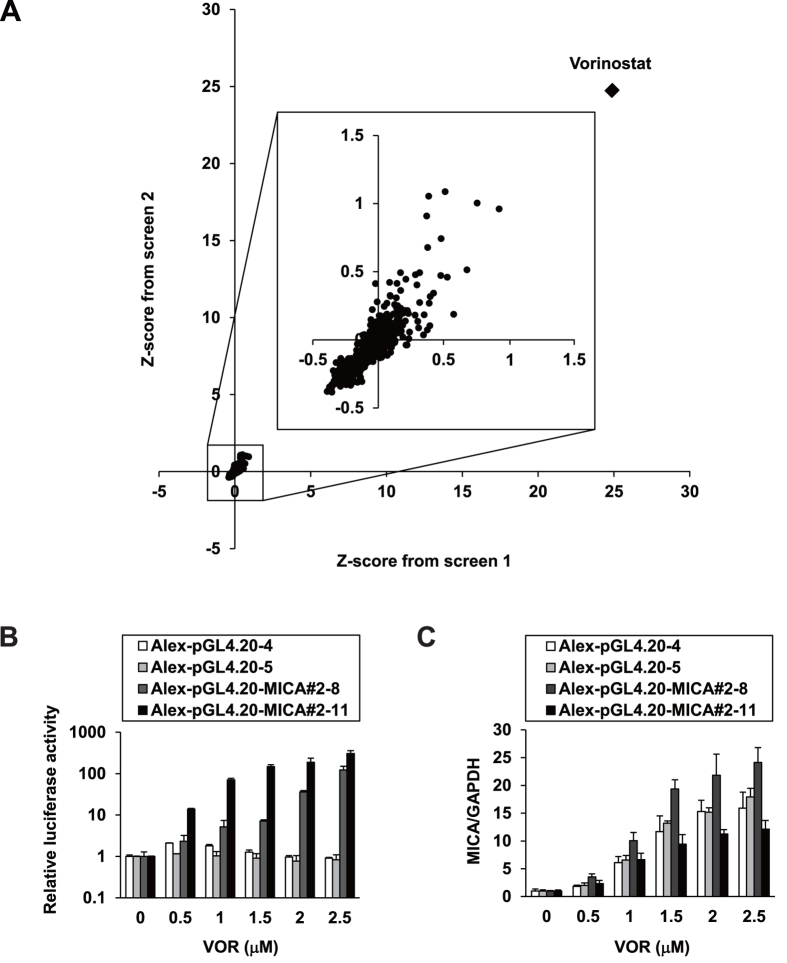
The screen for *MICA* expression inducers. (**A**) Alex-MICA#2-8 cells were treated with individual agents of the FDA-approved drug library for 48 h and lysed for a luciferase assay. Each dot represents the Z-score calculated from the fold-change of relative luciferase activity with normalization to cell viability, measured in duplicate. The Z-score of the top-hit VOR is indicated as a closed rhombus, and the plots of the other drugs are magnified in the large square in the middle. (**B**) The control cell clones Alex-pGL4.20-4 and -5 and the reporter cell clones Alex-pGL4.20-MICA#2-8 and -11 were treated with VOR for 48 h, and lysed for a luciferase assay. Firefly luciferase activity was normalized to the cell viability determined by a tetrazolium salt assay immediately before cell lysis, yielding the relative luciferase activity. (**C**) Total RNA was extracted from the cell clones treated as described in (**B**) and *MICA* mRNA levels were quantified by qRT-PCR with normalization to *GAPDH*.

We next screened the FDA-approved drug library using the established reporter system to find clinical agents capable of inducing MICA expression. Among the 636 approved drugs tested in Alex-pGL4.20-MICA#2-8 cells, the anti-cancer agent VOR emerged as the overwhelmingly strongest hit (Fig. 3A). We next tested its effects independently, and found that the luciferase activities of Alex-pGL4.20-MICA#2-8 and -11 were significantly increased in a dose-dependent manner (Fig. 3B), with accompanying increases in *MICA* mRNA levels (Fig. 3C). These results validated VOR as a potent inducer of MICA expression.

### Selective induction of MICA expression in HCC cells

**Figure 4:**
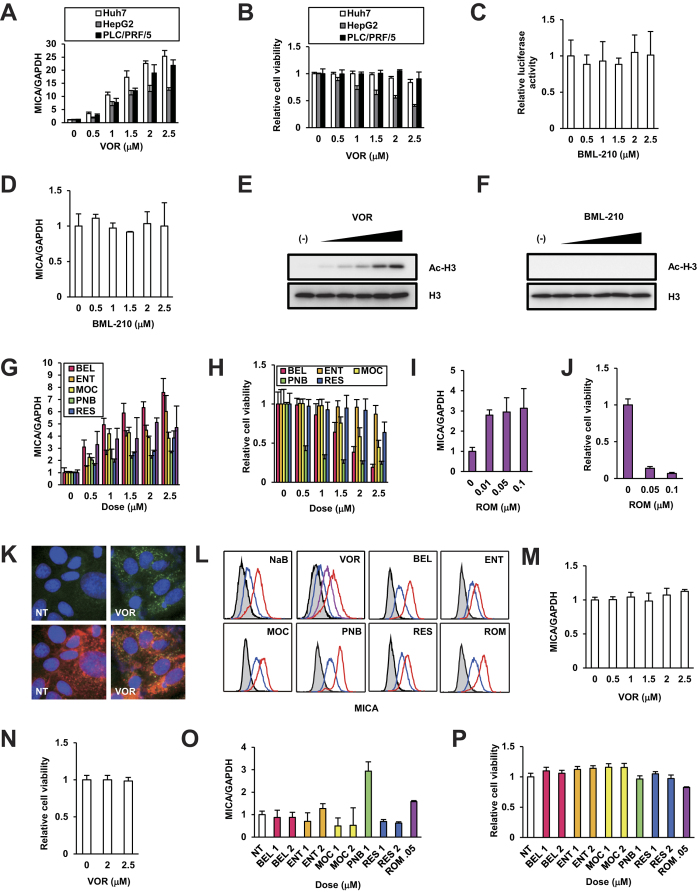
HDACi selectively upregulated *MICA* expression in HCC cells. (**A**) PLC/PRF/5 as well as Huh7 and HepG2 cells were treated with VOR for 48 h and relative *MICA* mRNA levels were quantified by qRT-PCR with normalization to *GAPDH*. (**B**) As in (**A**), the three cell lines were treated with VOR, and cell viabilities were determined by a tetrazolium salt assay. Alex-pGL4.20-MICA#2-8 (**C**) and naïve PLC/PRF/5 cells (**D**) were treated with BML-210 for 48 h at the indicated concentrations and relative luciferase activities normalized to cell viabilities and MICA mRNA levels normalized to *GAPDH* were determined. PLC/PRF/5 cells were treated with VOR (**E**) or BML-210 (**F**) at 0.5, 1, 1.5, 2, and 2.5 μM, detecting total and acetyl histone H3. PLC/PRF/5 cells were treated with BEL, ENT, MOC, PNB, RES (**G** and **H**), and ROM (**I** and **J**) at the indicated concentrations for 48 h, followed by quantification of relative *MICA* mRNA levels by qRT-PCR with normalization to *GAPDH* and relative cell viabilities. (**K**) After 48 h treatment with VOR at 2 μg/mL, PLC/PRF/5 cells were fixed and stained green with the antibody against MICA (upper panels), and merged with the plasma membrane shown in red (lower panels). (**L**) PLC/PRF/5 cells treated with either NaB (0 and 4 mM in blue and red, respectively), VOR (0, 1, and 1.5 μM in blue, purple, and red, respectively), BEL (0 and 2 μM in blue and red, respectively), ENT (0 and 2 μM in blue and red, respectively), MOC (0 and 2 μM in blue and red, respectively), PNB (0 and 0.5 μM in blue and red, respectively), RES (0 and 2 μM in blue and red, respectively), or ROM (0 and 0.05 μM in blue and red, respectively) for 48 h were immunolabeled with anti-MICA antibodies and fluorescent signals were detected by flow cytometry analysis, with the isotype controls shown as gray histograms. PXB cells were treated with VOR (**M** and **N**), BEL, ENT, MOC, PNB, RES, and ROM (**O** and **P**) for 48 h, followed by quantification of relative *MICA* mRNA levels by qRT-PCR with normalization to *GAPDH* and relative cell viabilities.

We next examined MICA protein expression in hepatoma cells. Clinical concentrations of VOR upregulated *MICA* mRNA levels in naïve PLC/PRF/5 cells, the parental cell line of our reporter cells, as well as in Huh7 and HepG2 cells (Fig. 4A)^11^. No significant cytotoxicity was observed in these cell lines except for HepG2 cells (Fig. 4B). The remarkable effects of VOR on MICA expression implied the validity of HDAC inhibition. These specific effects were further supported by the observation that BML-210, a VOR analog lacking HDAC inhibitory activity, did not elevate *MICA* promoter activity in Alex-pGL4.20-MICA#2-8 (Fig. 4C) or *MICA* mRNA expression levels in PLC/PRF/5 cells (Fig. 4D), and also did not result in histone H3 acetylation, in contrast to VOR (Fig. 4E,F). Therefore, we further examined the potencies of the approved HDACis belinostat (BEL), panobinostat (PNB), and romidepsin (ROM), and the clinically tested HDACis entinostat (ENT), mocetinostat (MOC), and resminostat (RES). In PLC/PRF/5 cells, BEL, ENT, and MOC markedly elevated *MICA* mRNA levels, followed by PNB and RES (Fig. 4G). Slight, moderate, and significant cytotoxicities were demonstrated by ENT and RES, BEL and MOC, and PNB, respectively (Fig. 4H). ROM enhanced the expression of *MICA* at lower concentrations (Fig. 4I), exhibiting significant cytotoxicity (Fig. 4J). Furthermore, immunofluorescence analysis showed that MICA protein expression was elevated in the VOR-treated PLC/PRF/5 cells, and highly colocalized with the plasma membrane (Fig. 4K). This result was supported by flow cytometry of PLC/PRF/5 cells, which directly detected a dose-dependent increase in the cell-surface expression of MICA induced by HDACis (Fig. 4L), and MICA protein expression on the membrane was confirmed to be enhanced by HDACi.

Therefore, to ensure safety, the pharmacological upmodulation of MICA expression should be confined to HCC cells. To evaluate the specificity, we treated normal human hepatocytes, PXB cells isolated from chimeric mice with a humanized liver (PXB mice)^12^, with the HDACis, and found no or little alteration in the *MICA* mRNA expression level and cytotoxicity by treatment with VOR (Fig. 4M,N), BEL, ENT, MOC, or RES (Fig. 4O,P). However, PNB and ROM treatment did show cytotoxicity, indicating the potential need for harnessing at lower doses. Taken together, these results suggest that HDACis are capable of inducing membrane MICA (mMICA) expression selectively in HCC cells.

### Enhanced NK cell cytotoxicity toward HCC cells

**Figure 5:**
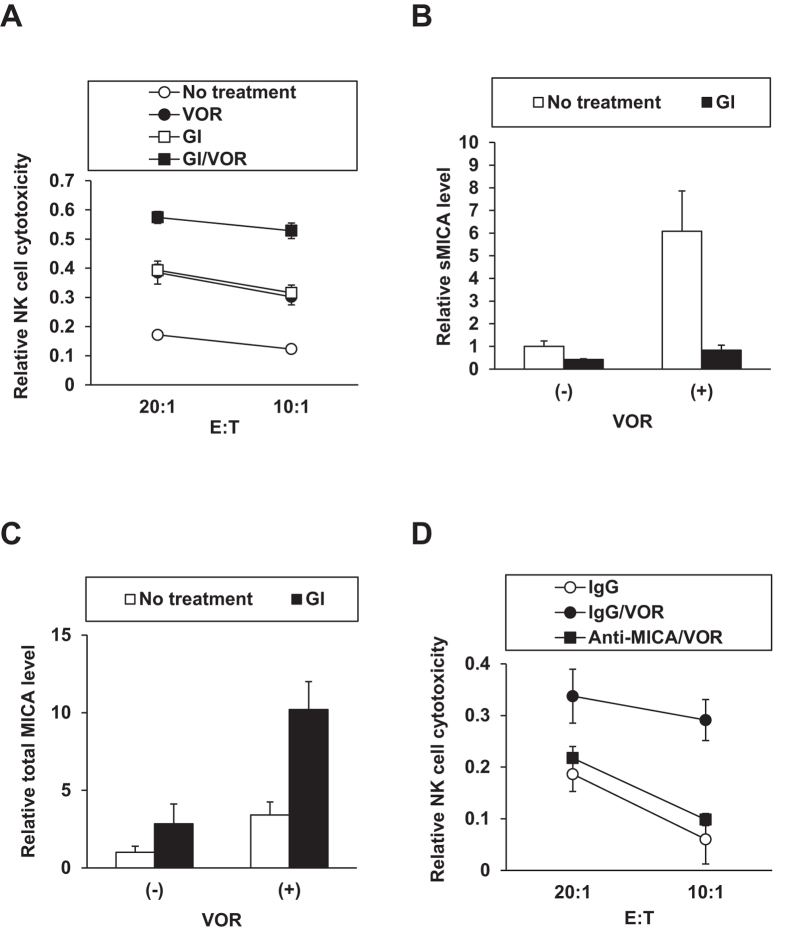
Enhanced NK cell cytotoxicity towards HCC cells through MICA. (**A**) PLC/PRF/5 cells pretreated with (closed symbols) or without (open symbols) VOR in the presence (squares) or absence (circles) of GI254023X (GI) for 24 h were cocultured with preprimed NK92MI cells at the indicated effector:target (E:T) ratios for 4 h, followed by measurement of the level of LDH release in the culture medium. Relative NK cell activities were calculated as described in the Methods. Also levels of soluble and total cellular MICA of PLC/PRF/5 cells treated in (**A**) were determined by ELISA in (**B**) and (**C**), respectively. (**D**) The assay was performed with (closed symbols) or without (open symbol) VOR pretreatment of PLC/PRF/5 cells in the same setting as described in (**A**) in the presence of control IgG (circles) or anti-MICA antibody (square), and analyzed as described above.

To evaluate the effect of HDACi-induced hepatocellular MICA expression on NK cell-mediated cytotoxicity, we employed a co-culture system. In brief, PLC/PRF/5 cells, which are relatively resistant to NK cells^13^, were pretreated with the representative HDACi VOR for 24 h to induce MICA expression, followed by co-incubation for 4 h with the preprimed NK cell line NK92MI as the effector cells, whose NKG2D expression level was diminished by VOR (Supplementary Figure S3A,B), consistent with previous observations^14^,^15^, with the cell viability unaltered (Supplementary Figure S3C) in a separate experiment. Then the level of lactate dehydrogenase (LDH) release in the co-culture medium was measured. At every effector:target (E:T) ratio, the relative level of LDH release from PLC/PRF/5 cells pretreated with VOR was significantly higher than that of untreated cells (Fig. 5A). These effects were further reinforced when the target cells were pretreated with the MICA shedding inhibitor GI254023X^16^, which decreased the soluble MICA (sMICA) level (Fig. 5B) and increased the total MICA protein level (Fig. 5C) in proportion to changes in the mMICA level (data not shown). Next, we evaluated whether the enhancement of NK cell cytotoxicity is mediated by MICA using an anti-MICA antibody capable of blocking the MICA–NKG2D interaction. Enhancement of specific LDH release from the PLC/PRF/5 cells with upregulated MICA expression was detected in the presence of the control antibody IgG, which was abrogated by treatment with the anti-MICA antibody (Fig. 5D). Thus, induction of mMICA expression by HDACi in HCC cells was confirmed to enhance NK cell cytotoxicity through MICA signaling, leading to the more efficient elimination of target tumor cells.

### *In vivo* anti-tumor responses to human HCC in an NKG2D-dependent manner

**Figure 6:**
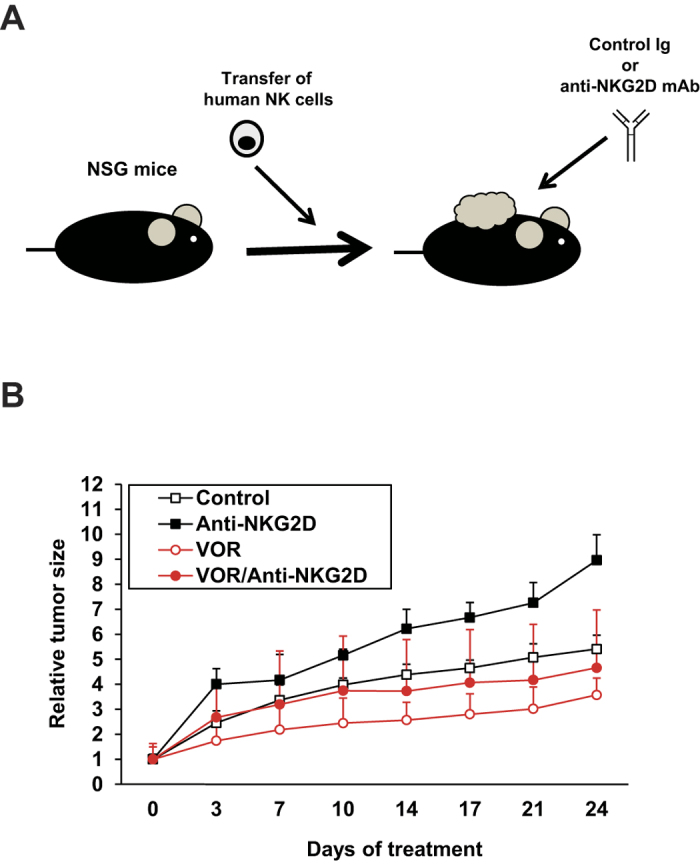
The NKG2D immune system regulates the anti-tumor activities of VOR in an *in vivo* humanized animal model. (**A**) Schema of the *in vivo* experimental design. (**B**) CD56+ NK cells isolated from PBMCs obtained from a healthy volunteer were transferred intravenously into the NSG mice (n = 5 per group) along with subcutaneous administrations of PCL/PRF/5 HCC cells. After establishment of subcutaneous tumors, the mice were then treated with control Ig or anti-NKG2D monoclonal antibody (1 mg/kg) twice per week. The relative tumor growth kinetics in each mouse was evaluated on the indicated days.

We further evaluated the impact of MICA-NKG2D pathways on the *in vivo* tumorigenicity of HCC. For this purpose, PCL/PRF/5 cells were injected subcutaneously into immunodeficient NSG mice, and treated with control Ig or an anti-NKG2D monoclonal antibody that specifically interferes with the human NKG2D–ligand interaction^17^. During this procedure, the adoptive transfer of NK cells from healthy donors (2 × 10^6^ cells/mice) was performed via intravenous injection (Fig. 6A). VOR treatment significantly suppressed tumor growth, whereas treatment with anti-NKG2D promoted the growth of PLC/PRF/5 tumors (Fig. 6B and Supplementary Table S2). Moreover, anti-NKG2D treatment largely abrogated the anti-tumor effect against PLC/PRF/5 tumors (Fig. 6B and Supplementary Figure S4). Overall, these findings provide evidence that the NKG2D-mediated regulation of NK cell activities serves as a critical pathway for controlling the anti-tumor effect of HDACi against human HCC cells.

### Metabolomics uncovered the physiological modes of the HDACi chemoimmunotherapeutic action

**Figure 7:**
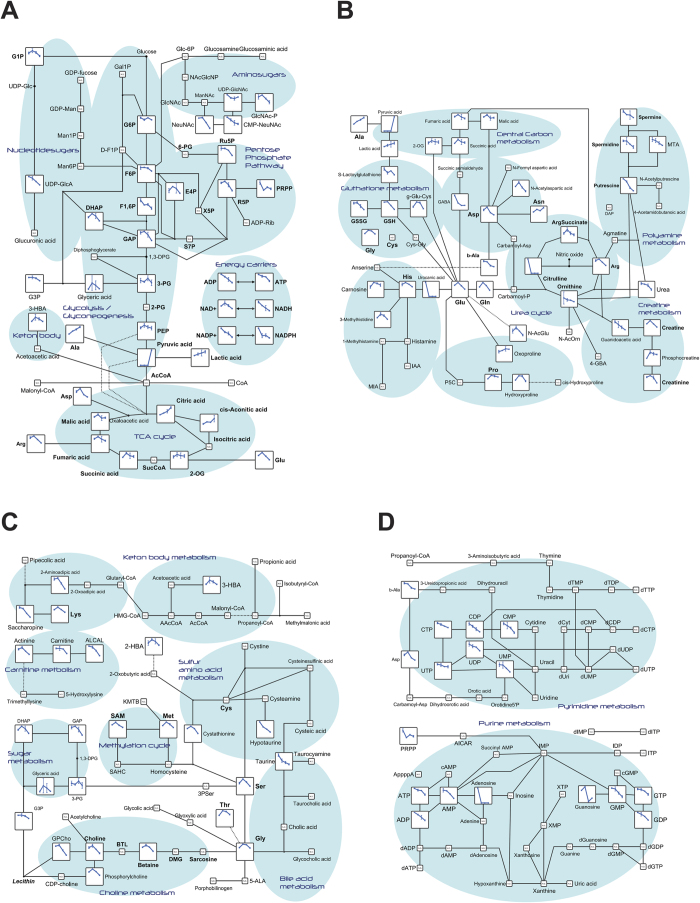
Metabolic alterations in HDACi-treated HCC cells. The relative levels of detected metabolites in PLC/PRF/5 cells are mapped in the indicated metabolic pathways including central carbon metabolism (**A**), urea cycle and related amino acid metabolism (**B**), lipid and related amino acid metabolisms (**C**), and nucleic acid metabolism (**D**), as line graphs at 0, 24, 48, and 72 h post-treatment with VOR. The pathway analysis was performed using Visualization and Analysis of Networks containing Experimental Data^65^. N.D., not detected.

Finally, we sought to mechanistically decipher the physiological modes of the HDACi therapeutic action, and conducted metabolomics analysis on PLC/PRF/5 cells treated with 2 μM VOR by capillary electrophoresis time-of-flight mass spectrometry (CE-TOFMS). Of the 162 metabolites detected, 75 were quantified and mapped to major metabolic pathways as primary factors. The most notable finding was the alteration in central carbohydrate metabolism (Fig. 7A), in which the activities of the glycolytic pathway at early stages and the pentose phosphate pathway were consistently reduced. This result is in line with the elevated expression of retinoblastoma protein (pRb) and phosphatase and tensin homolog (PTEN) (Supplementary Figure S5A), which are tumor suppressor genes that reportedly reduce glycolysis in cancer cells^18^. However, the metabolism of pyruvic acid appeared to operate effectively, as detected by the elevated levels of alanine and lactic acid, which stimulated MICA expression (Supplementary Figure S5B). By contrast, in the tricarboxylic acid (TCA) cycle, citric and cis-aconitic acids accumulated, and ATP-citrate lyase (ACLY) appeared to facilitate MICA induction by VOR (Supplementary Figure S5C). In coordination with these observations, the amount of energy carriers decreased, confirming the overall diminished energy supply.

The urea cycle and related pathways also exhibited evident changes following treatment with VOR (Fig. 7B). The level of total glutathione (GSH) dropped continuously, and the amounts of multiple amino acids such as glutamic acid (Glu), glutamine (Gln), aspartic acid (Asp), and arginine (Arg), as well as intermediates such as gamma-aminobutyric acid (GABA), urocanic acid, citrulline, and ornithine, eventually decreased in and around the urea cycle. Harmoniously, polyamide and creatinine were also depressed, showing general deficiency in energy storage and a supply for HCC cell proliferation.

Similarly, lipids and related amino acid metabolism was downregulated (Fig. 7C). Specifically, the pathway activities of carnitine, choline, and lysine (Lys), and the level of serine (Ser) conspicuously tended to decline, particularly after 24 h, reflecting the treatment-initiated attenuation of related chain reactions, while taurine levels were consistently reduced.

Nucleic acid metabolism was globally downregulated as well (Fig. 7D). The total adenylate and guanylate levels were grossly reduced, although the adenylate and guanylate energy charge levels remained unaltered (data not shown), indicating impairment of nucleic acid synthesis. In addition, the activities of nicotinamide and coenzyme (CoA) metabolic pathways were reduced (Supplementary Figure S1), whereas no significant trends were recognized in the metabolism of branched-chain and aromatic amino acids (Supplementary Figure S2).

## Discussion

Our study specified the additional effect of an approved drug in robustly upregulating the expression of the HCC susceptibility gene identified in genome-wide exploration, and thereby boosting the anti-HCC effects of NK cells. Concomitantly novel insights into the cellular physiological modes of actions were obtained based on analysis of the metabolic alterations during the treatment with the drug. These results provide not only the proof of concept for novel pharmacotherapeutic strategies to inhibit HCC but also provide a further mechanistic explanation of the immunoactivating ligand regulation and the associated chemotherapeutic effects of HDACi.

MICA is the key ligand for anti-tumor immunity, activating NKG2D-bearing lymphocytes such as T and NK cells, and its expression is induced in cells under stresses typified by malignant transformation for immune surveillance^19^, whose impairment has been observed in chronic liver disease with viral infection leading to HCC. In HCV-infected cells, mMICA expression was reported to be downregulated via NS3/NS4A^20^, NS2, and NS5B^21^. Furthermore, the risk allele of rs2596538, a single nucleotide polymorphism in the *MICA* promoter sequence that causes HCC, was associated with lower sMICA levels in HCC patients^22^. Likewise, mMICA expression was diminished in HBV-producing HepG2.2.15 cells^23^, and HBV inhibition restored MICA expression, rendering hepatoma cells susceptible to NK cells^24^. Suppression of MICA in HCC cells overexpressing the HBV surface antigen also reduced their sensitivity to NK cells^25^. Furthermore, MICA expression was reduced in a tumor-node-metastasis stage-dependent manner and correlated with relapse-free and/or overall survival rates in HBV-HCC patients^26^,^27^. These facts are in agreement with our previous GWAS results suggesting that MICA and the NKG2D system are critical for proper anti-tumor immunity in chronic hepatitis virus infection, and hence restoration of MICA expression is a feasible treatment strategy for HCC to overcome its molecular complexity^3^.

Concurrent with our discovery and analyses, a cell biological study mechanistically endorsed the upregulation of MICA expression by VOR, at least in cultured hepatoma cells^28^. As a pan-HDACi, recognizing both class I (HDAC1, HDAC2, and HDAC3) and class II (HDAC6) HDACs^11^, VOR was shown to acetylate the histones associated with the *MICA* promoter, thereby enhancing the transcription^28^. Indeed, the significance of HDAC inhibition as a mode of MICA upmodulation in hepatoma cells has been demonstrated for other agents, including NaB^8^, valproic acid (VPA)^8^,^29^, and ENT^30^. In addition, HDACi-mediated acetyl histones associated with the *MICA* promoter^31^,^32^ were identified in various tumors, supporting the classic action of HDACis in hepatoma cells. Furthermore, our reporter system helped to determine the transcription factors mediating the induction of MICA expression. To date, several molecules have been implicated in the mechanism of action of this HDACi (Supplementary Table S1), and we are currently investigating critical candidates as new intervention targets.

Mechanistically, the metabolomics analysis offered novel and significant insights into the physiological events underlying the HDACi-enhanced expression of MICA. In accordance with reports in other types of cancer cells treated with HDACis^33^,^34^, the glycolytic pathway was found to be primarily deactivated during VOR treatment (Fig. 7A). Promotion of the glycolytic pathway is known as the Warburg effect, providing efficient cellular energy production in cancers including HCC^35^. Therefore, this phenomenon of glycolysis reduction was presumably associated with the induction of the expression of tumor suppressor genes that reduce glycolysis (Supplementary Figure S5A), and inhibition of metabolic oncogenes, as exemplified by hypoxia inducible factor1α in Huh7 cells^36^. Moreover, the glycolysis was possibly halted at late stages, as deduced from the accumulated Ala and lactic acid from pyruvic acid. Intriguingly, lactic acid found to inhibit the HDAC^37^ induced the expression of MICA (Supplementary Figure S5B) as observed in Jurkat T cells^38^. By contrast, metabolites in the TCA cycle remained generally constant, which was possibly fueled by the consumption of amino acids such as Arg, Asp, and Glu, except that citric and cis-aconitic acids quantitatively increased. Acetyl-CoA is used for lysine acetylation, which is generated by ACLY from citrate^39^, suggesting a potential contribution of citrate to MICA expression via histone acetylation (Supplementary Figure S5C). The demonstrated energy depletion causes energy stress, which consequently stimulates mitochondrial oxidative phosphorylation for energy compensation. However, the level of total GSH was reduced along with the diminishing quantity of taurine, which conceivably exacerbated oxidative stress. These multiple and amplified stresses are also thought to upregulate the expression of MICA, a stress-induced gene. Thus, these molecules and pathways could help to explain the underlying mechanism of HDACi and become targets for the enhancement of MICA expression.

Overall, these results suggested that glycolytic pathway inhibition and mitochondrial operation mediated by tumor suppressor genes (Supplementary Figure S5A), and accumulation of lactic acid and citric acid (Fig. 7A) with phosphofructokinase 1 in feedback loops^40^, as well as the decreased levels of GSH and taurine are all disadvantageous for HCC cell proliferation in terms of energy demands and oxidative stress. In addition, we identified novel anti-HCC features of HDACi. Specific pathways for energy supply and storage, such as the metabolism of polyamine, creatinine (Fig. 7B), ketone body, carnitine (Fig. 7C), and CoA (Figure S1), were downregulated. Furthermore, the decline in the levels of amino acids is known to support tumor growth, as exemplified by Gln^41^ and Ser^42^ and nucleic acid synthesis (Fig. 7D), which is in line with disturbed HCC cell proliferation. Thus, HDACi was indeed exhibited to exert anti-cancer effects through energy depletion and apoptotic cell stresses. Therefore, besides the direct efficacy of HDACi chemotherapy, the eventual elimination of anti-apoptotic HCC cells by NK cells via targeting MICA could be expected to ultimately achieve efficient chemoimmunotherapy (i.e., the combination of targeted chemotherapy and immunotherapy).

In practice, the pharmacological augmentation of cancer immunity via NKG2DL has been successfully implemented. For example, administration of all-*trans*-retinoic acid or VPA upregulated MICA expression in myeloid leukemic cells of patients, causing efficient cell lysis by autologous CD8+ T and NK cells^43^. Therefore, HDACis could be valuable agents for the immunological control of HCC, especially in postoperative therapy such as in adjuvant interferon therapy for HCV-HCC^44^ where recurrence is frequent^45^. This would be expected to be even more effective in individuals carrying the rs2596542 polymorphism, which predicts an increased genetic risk of insufficient MICA induction^5^. Indeed, VOR induced MICA expression in Huh7 cells with the risk genotype AA as well as in PLC/PRF/5 and HepG2 cells with the AG genotype (data not shown).

The anti-cutaneous T cell lymphoma (CTCL) drug VOR has been recognized to inhibit the proliferation of various tumor cell types, including HCC, in xenograft mouse models^46^,^47^,^48^,^49^. Besides its direct anti-tumor effect, our *in vivo* analysis newly testified to its immunological efficacy through NKG2D signaling, although the impacts of other pathways cannot be ruled out. This enhances the attractiveness of the dual effectiveness of HDACis^50^, as recently perceived through the immunomodulatory effects of anti-cancer drugs^51^. Preceded by BEL demonstrating tumor stabilization in patients with unresectable HCC^52^, HDACis are expected to be employed for HCC treatment as a substitute for or in combination with sorafenib, the only currently approved agent for advanced HCC^53^. Furthermore, the particular effectiveness for HCC cells harboring wild-type p53 represented by HepG2 cells^54^, which are relatively susceptible to VOR (Fig. 4B) through p53^55^, would be desirable. HDACis are thus assumed to constitute chemoimmunotherapy with strategic evaluations^56^.

Safety considerations are required, as excessive tumor immunity could lead to the development of an autoimmune disease, and NKG2DL-NKG2D signaling is no exception^57^. For selective immunoactivation, the HCC cell-specific induction of MICA expression by VOR is favorable. Similarly, the narrow-spectrum HDACi ENT elevated the expression level of NKG2DLs in colon cancer cells without affecting normal cells *in vivo*, demonstrating its utility in NK-cell immunotherapy for solid tumors^32^. Mechanistically, the cancer cell-selective induction of MICA could be ascribed to multiple targets potentially encompassing HDAC status and cancer cell metabolism, and is as important as the cancer-specific apoptosis by HDACi^58^. Concurrently, sMICA is known to evade NKG2D-mediated immunosurveillance^6^, and therefore MICA upregulation ought to be implemented carefully when sMICA production could be promoted^59^. In actuality, the addition of a MICA sheddase inhibitor reinforced the evoked mMICA expression and NK cell cytotoxicity (Fig. 5A) as observed in various cancer cells^60^. Additional drugs would be appropriately administered in combination with HDACi to mitigate any imbalance. With regard to the chemical safety, clinical HDACis should be advantageous, because the pharmacokinetic properties have been well documented as represented by VOR^11^. Further investigations would ensure safe and preferable methods and compounds, with effects on NKG2D in NK cells also taken into account, as indicated by the difference between ENT and pan-HDACis^32^.

Cancer immunotherapy has received increased attention recently, and was deemed the “scientific breakthrough of the year” in 2013 by *Science*^7^. In a remarkable example, the blockade of immune checkpoints represented by cytotoxic T lymphocyte antigen 4 and programmed death 1 was shown to be efficacious for melanoma patients and was recently approved for diverse cancer types^61^; it is presently in clinical trials for HCC^62^. Thus, interference with the ligand-receptor system in immunity is becoming more widely accepted as a promising method for cancer treatment. In this regard, the major NKG2DL MICA is emerging as a prospective target^19^ by virtue of its tumor specificity. Pragmatically, approved and additional HDACis could become feasible clinical chemoimmunotherapeutic options, considering the genetic features of individual patients^5^ for more personalized and selective medicine^59^. Finally, new avenues for the future of promising HCC innate immunotherapy would be opened by GWAS-based drug development.

## Methods

### Compounds and cells

VOR, and NaB and puromycin were purchased from Cayman Chemical (Ann Arbor, MI, USA) and Wako Pure Chemical (Osaka, Japan), respectively. BML-210, GI254023X, sodium DL-lactate (NaL), and BMS-303141 were purchased from Sigma-Aldrich (St. Louis, MO, USA). BEL, ENT, MOC, PNB, and RES were purchased from Selleck Chemicals (Houston, TX, USA), and ROM was purchased from Abcam (Cambridge, United Kingdom). Antibodies to histone H3 and acetyl-histone H3 were purchased from Cell Signaling Technology (Danvers, MA, USA). IL-15 and Cell Counting Kit-8 were purchased from R&D Systems (Minneapolis, MN, USA) and Dojindo (Kumamoto, Japan), respectively. Huh7 and HepG2 cells, as well as PLC/PRF/5 and the NK cell line NK92MI were obtained from American Type Culture Collection (Manassas, VA, USA), and were cultured according to the supplier’s protocols. The cell lines were authenticated by the short tandem repeat method (Bex, Tokyo, Japan) in January 2016. PXB cells were purchased from Phoenix Bio (Hiroshima, Japan).

### FDA-approved drug screen

Alex-pGL4.20-MICA#2-8 cells were treated with drugs in the FDA-Approved Drug Screen-well Library (Enzo Life Sciences, Farmingdale, NY, USA) for 48 h, and the cell viabilities were determined with Cell Counting Kit-8. The firefly luciferase activities were measured as described previously^63^ and normalized to the cell viabilities to obtain the fold-change in the efficacy for each drug compared with the untreated control. Z-scores were then calculated by dividing the difference between each comparison and median fold-changes based on the standard deviation of all the wells in the plate.

### Quantitative reverse transcription-polymerase chain reaction

Relative mRNA levels of individual genes were quantified as previously described^63^ using the following primer sets: MICA-F 5′-CTTCCTGCTTCTGGCTGGCATC-3′ and MICA-R 5′-CAGGGTCATCCTGAGGTCCTTTC-3′ for *MICA*; pRB-F 5′-CTCTCGTCAGGCTTGAGTTTG-3′ and pRb-R 5′-GACATCTCATCTAGGTCAACTGC-3′ for *pRB*; PTEN-F 5′-AGGGACGAACTGGTGTAATGA-3′ and PTEN-R 5′-CTGGTCCTTACTTCCCCATAGAA-3′ for *PTEN*; NKG2D-F 5′-GAGTGATTTTTCAACACGATGGC-3′ and NKG2D-R 5′-ACAGTAACTTTCGGTCAAGGGAA-3′ for *NKG2D*; and GAPDH-F 5′-ATGGGGAAGGTGAAGGTCG-3′ and GAPDH-R 5′-GGGGTCATTGATGGCAACAATA-3′ for glyceraldehyde-3-phosphate dehydrogenase (*GAPDH*), with the value of *MICA* normalized to that of *GAPDH*.

### Plasmid

The luciferase reporter vector pGL4.20 was purchased from Promega (Madison, WI, USA). The promoter sequences of *MICA* encoded on pLightSwitch_Prom (SwitchGear Genomics, Menlo Park, CA, USA) were amplified by the primers EcoRV-MICA-gDNA-F3 5′-ATCGATATCGTGGGATTGAAATAGCGTTGAAG-3′ and HindIII-MICA-gDNA-R2.1 5′-ATCAAGCTTGGAGGTGCAAAAGGGAAGATG-3′, and subcloned into pGL4.20, producing pGL4.20-MICA#2.

### Luciferase assay

Firefly luciferase activity was monitored by a dual-luciferase reporter assay system (Promega) as described previously^63^, and normalized to *Renilla* luciferase activities from pRL-TK, or the total protein concentration of cell lysates was quantified using the Bradford protein assay reagent (Bio-Rad, Hercules, CA, USA).

### Reporter cell clone

Cells of the human hepatoma cell line PLC/PRF/5 were transfected with pGL4.20 or pGL4.20-MICA#2 and colonies harboring the plasmid were selected for 2–3 weeks in the presence of 7 μg/mL puromycin. Subsequently, the surviving clones were isolated and propagated individually.

### Immunofluorescence

Cells were fixed in 4% paraformaldehyde and immunostained with a rabbit anti-MICA polyclonal antibody (Abcam, Cambridge, UK), followed by the Alexa 488-conjugated anti-rabbit antibody (Life Technologies, Rockville, MD, USA) with Alexa 594-conjugated wheat germ agglutinin (Life Technologies), for labeling plasma membranes, and the nuclear dye Hoechst 33258 (Dojindo). Fluorescent images were obtained with Floid Cell Imaging Station (Life Technologies).

### Western blotting

Total protein was resolved by SDS-PAGE and subjected to western blotting as described previously^63^.

### Flow cytometry

Suspended hepatoma and NK cells were incubated with the following antibodies (R&D Systems) according to the manufacturer’s protocol: Alexa 488-conjugated mouse IgG2B isotype control, Alexa 488-conjugated human MICA antibody, or Alexa 488-conjugated human NKG2D antibody. Fluorescent signals were detected with BD Accuri C6 (BD Biosciences, San Jose, CA).

### ELISA

MICA protein in culture supernatants and whole cell lysates of PLC/PRF/5 cells were detected by MICA ELISA Kit (Diaclone, Besançon, France) according to the manufacturer’s protocol.

### NK cell cytotoxicity assay

NK cell-mediated cytotoxicity toward target hepatoma cells was determined with an LDH cytotoxicity detection kit (Takara Bio, Shiga, Japan) according to the manufacturer’s protocol. In brief, NK92MI cells were primed with 50 ng/mL IL-15 for 24 h and PLC/PRF/5 cells were pretreated with 1.5 μM VOR and/or 50 μM GI254023X, and were co-cultured at several E:T ratios for 4 h, followed by measurement of LDH release in the supernatants. The NK cell cytotoxicity was calculated with the following formula: Cytotoxicity (%) = 100 × [(Effector: Target cell mix − Effector cell control) − Low control]/(High control − Low control). For the high control, 0.5% Triton X-100 (Sigma-Aldrich) was used. In the blocking assay, the cells were incubated with either mouse IgG2B isotype control or human MICA antibody (R&D Systems) at 10 μg/mL.

### *In vivo* experimental design

To examine the *in vivo* anti-tumor responses induced by VOR and anti-NKG2D monoclonal antibodies, PLC/PRF/5 cells were inoculated subcutaneously into severe immunodeficient NSG mice (1 × 10^6^ cells/mouse) in conjunction with intravenous administration of CD56+ NK cells obtained from healthy volunteers (2.5 × 10^6^ cells/mouse). The blood mononuclear cells were obtained with informed consent in accordance with the protocols approved by the review board of Hokkaido University [14-0002 (1)]. The animal protocol was approved by the Institutional Animal Care and Use Committee of the National University Corporation, Hokkaido University [14-0025]. All experiments were performed in accordance with relevant guidelines and regulations. After establishment of subcutaneous tumors, the mice were treated with intraperitoneal injections of control Ig or the anti-NKG2D monoclonal antibody (clone HMB-1: 1 mg/kg) twice per week with or without VOR at 25 mg/kg/day. Tumor-bearing mice survived for over 30 days without treatment, and for 45 days with human NK cells. Tumor growth was measured on the indicated days. The means of actual tumor sizes with the standard deviations (SD) were calculated (Supplementary Table S2). The relative tumor growth kinetics were determined by calculating the percentage of the growing tumor volume relative to the initial tumor volume (Fig. 6B). The median values of the relative tumor sizes were demonstrated by box plots (Supplementary Figure S2).

### Metabolomics analysis

Metabolomics analysis was performed at Human Metabolome Technologies (Yamagata, Japan)^64^. In brief, PLC/PRF/5 cells were washed with 5% mannitol solution and permeated by LC/MS grade methanol (Wako) containing Internal Standard Solution 1 (HMT) at room temperature. After ultrafiltration of the extracts, metabolomics analysis was performed by CE-TOF/MS, followed by normalization of the signals to the cell number.

### Statistical analysis

The data from cell-based assays are presented as means ± SD.

## Additional Information

**How to cite this article**: Goto, K. *et al*. Novel chemoimmunotherapeutic strategy for hepatocellular carcinoma based on a genome-wide association study. *Sci. Rep.* **6**, 38407; doi: 10.1038/srep38407 (2016).

**Publisher's note:** Springer Nature remains neutral with regard to jurisdictional claims in published maps and institutional affiliations.

## Supplementary Material

Supplementary Information
