# Supplementary material for: Novel chemoimmunotherapeutic strategy for hepatocellular carcinoma based on a genome-wide association study
Source: Sci Rep. 2016 Dec 2;6:38407. doi: 10.1038/srep38407 (PMC5133582; doi:10.1038/srep38407)
Supplement: Supplementary Information [file srep38407-s1.pdf]

Novel chemoimmunotherapeutic strategy for hepatocellular carcinoma based on a genome-wide association study

Running title: GWAS-based HCC chemoimmunotherapy

Kaku Goto, Ph.D., Dorcas A. Annan, M.D., Tomoko Morita, Ph.D., Wenwen Li, M.D., Ph.D., Ryosuke Muroyama, M.D., Ph.D., Yasuo Matsubara, M.D., Ph.D., Sayaka Ito, Ryo Nakagawa, M.D., Yasushi Tanoue, M.D., Ph.D., Masahisa Jinushi, M.D., Ph.D., Naoya Kato, M.D., Ph.D.

Supplementary Information

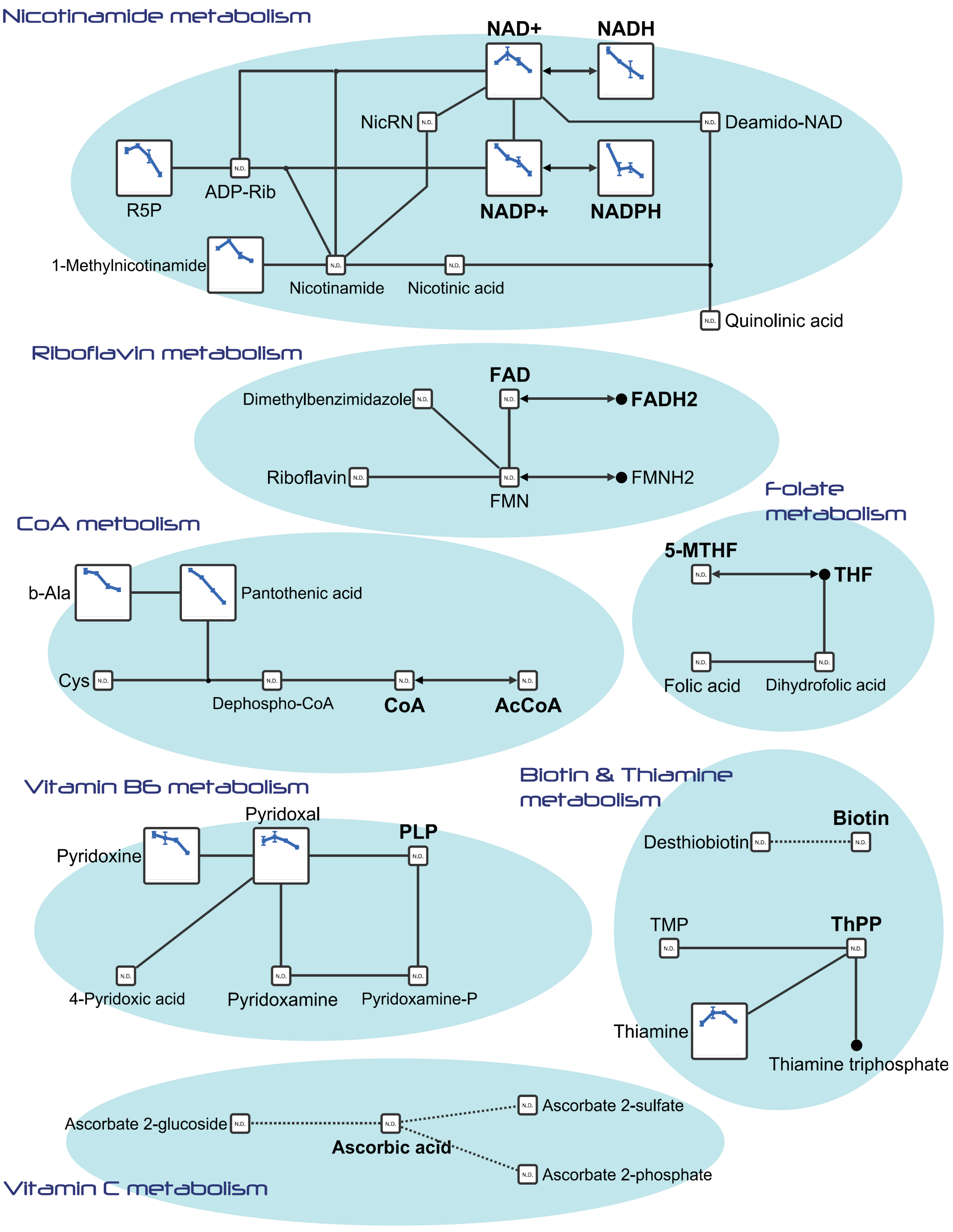

Supplementary Figure S1

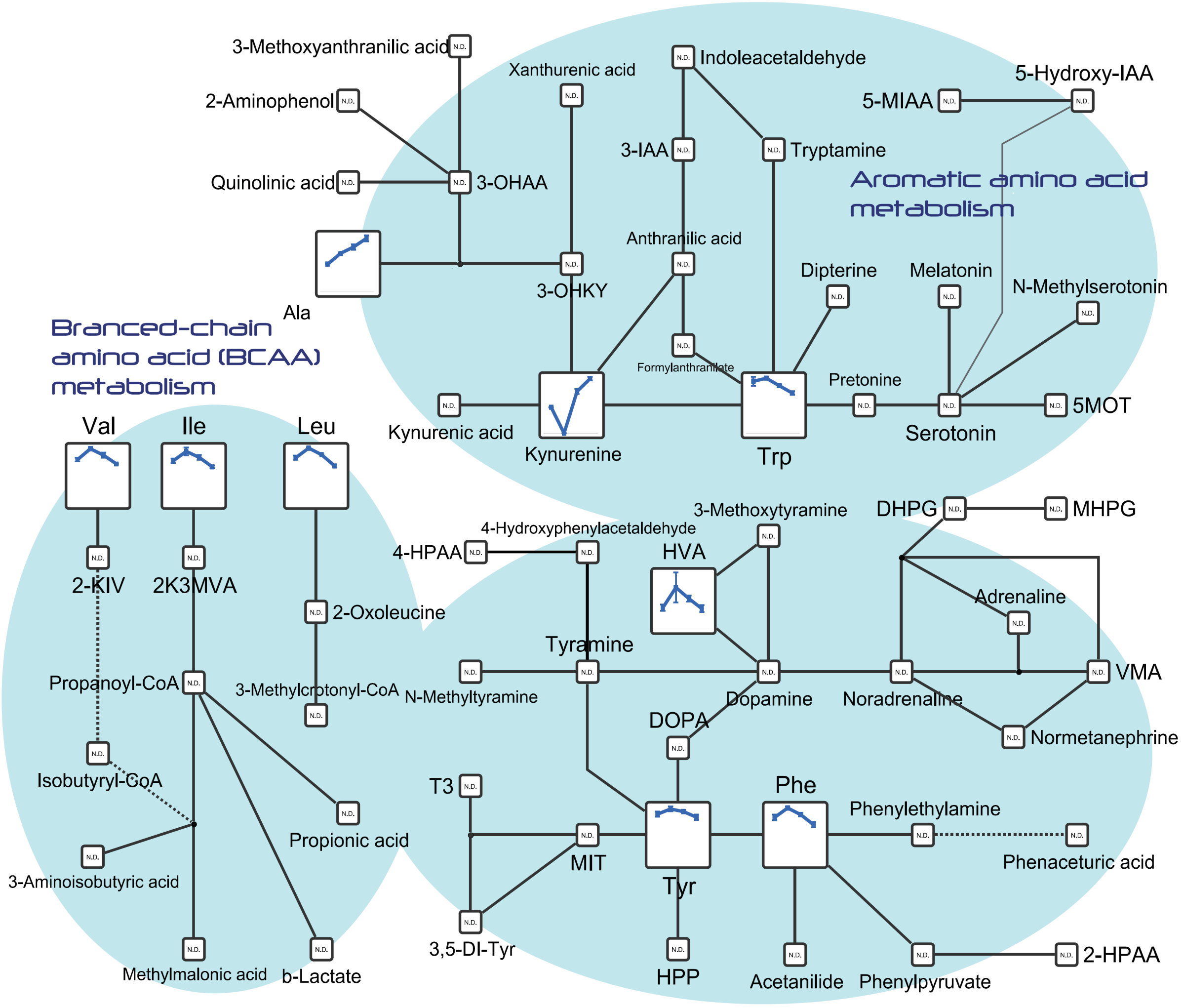

Supplementary Figure S2

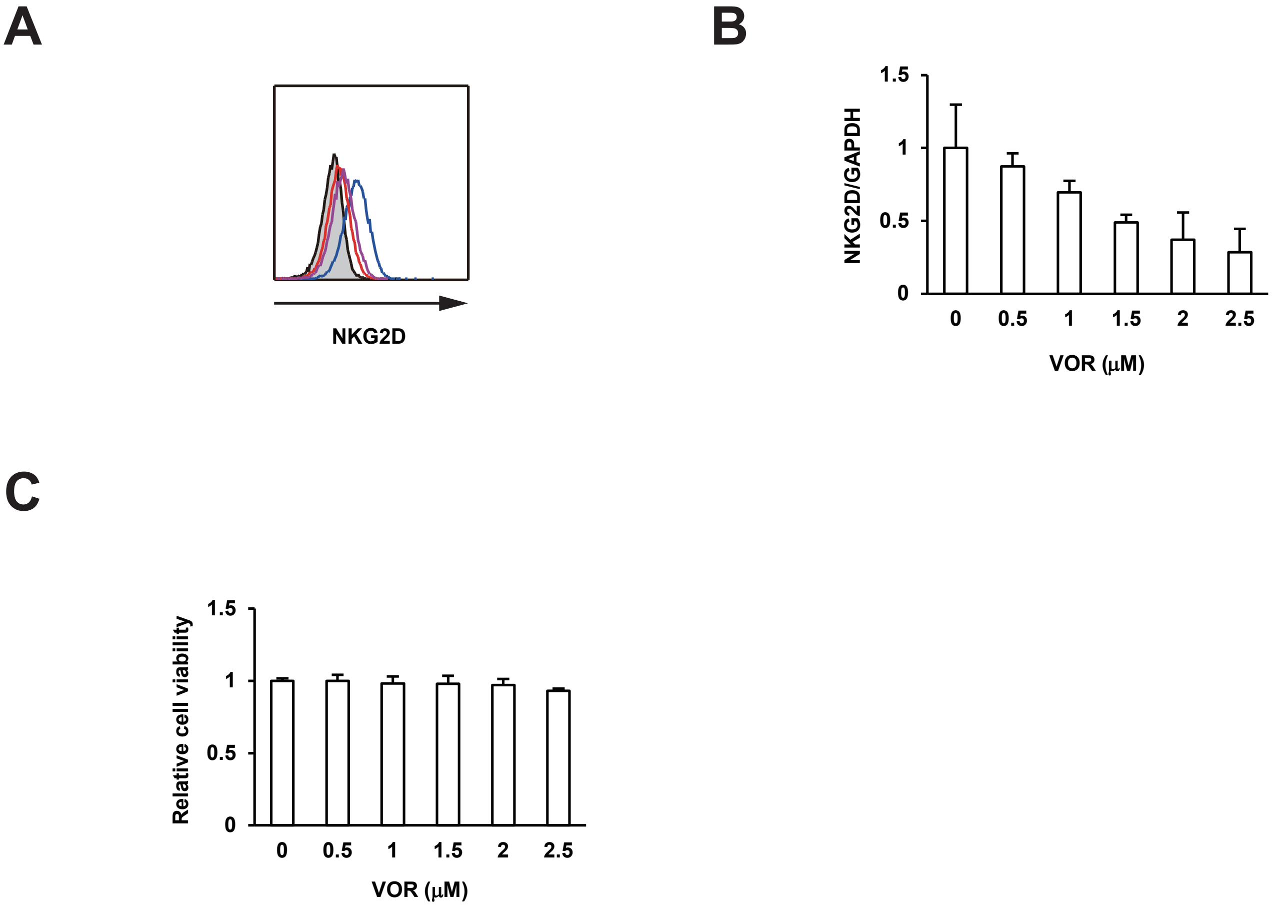

Supplementary Figure S3

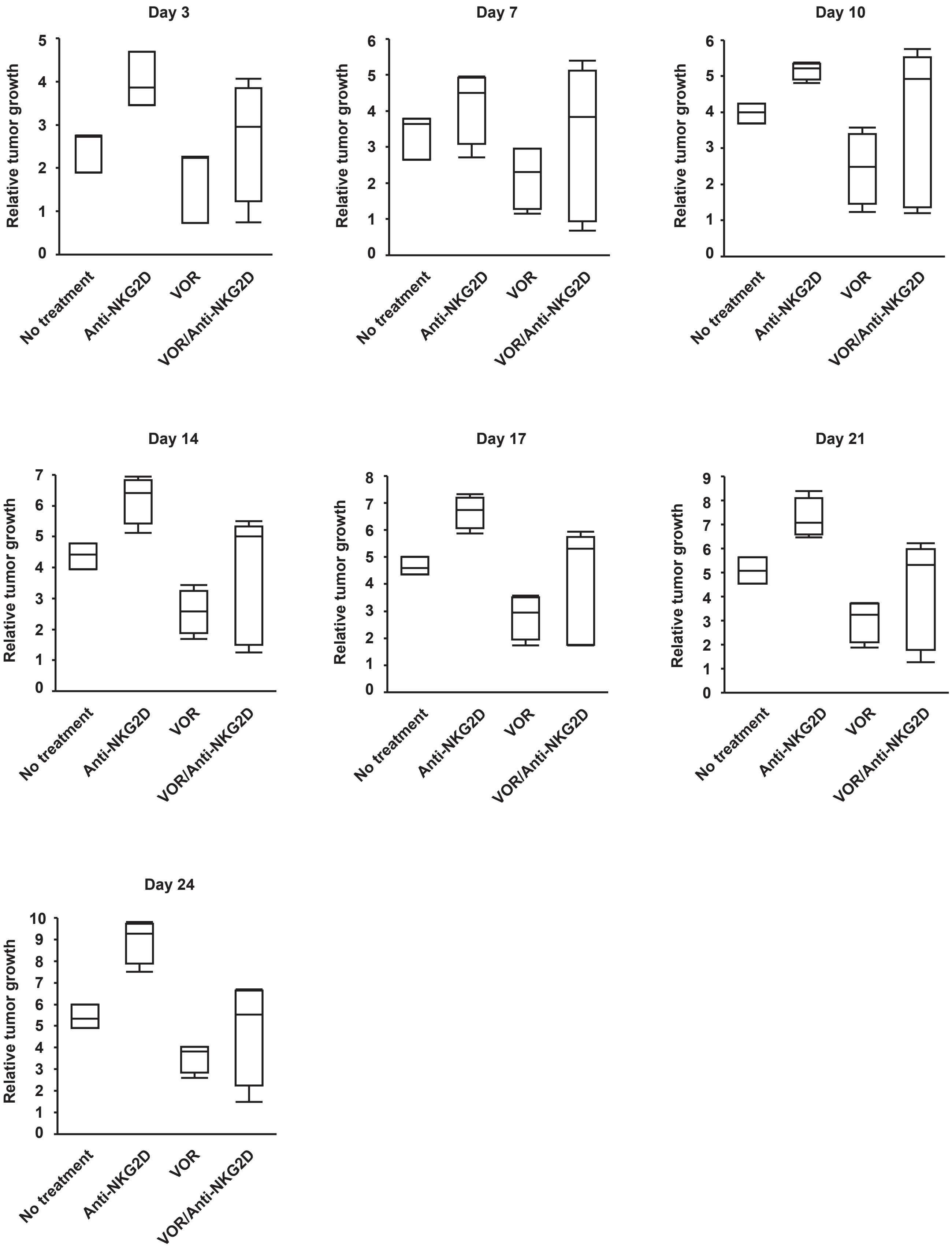

Supplementary Figure S4

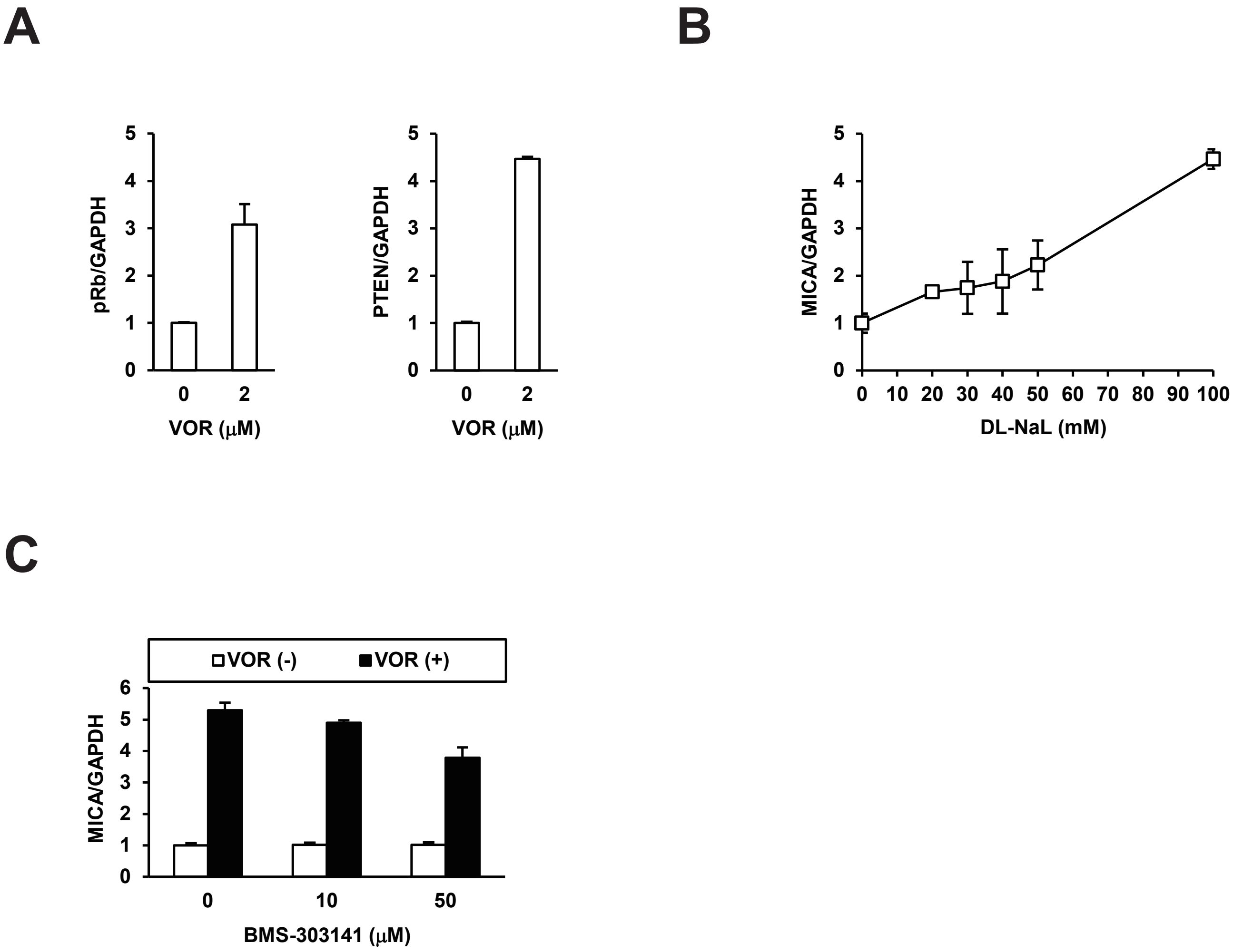

Supplementary Figure S5

Supplementary figure legends

Supplementary Figure S1. CoA metabolisms. The relative levels of detected metabolites are mapped in the indicated metabolic pathways as line graphs at 0, 24, 48, and 72 h post-treatment with VOR as in Figure 7. N.D., not detected.

Supplementary Figure S2. The metabolism of branched-chain and aromatic amino acids. The relative levels of detected metabolites are mapped in the indicated metabolic pathways as line graphs at 0, 24, 48, and 72 h post-treatment with VOR as in Figure 7. N.D., not detected.

Supplementary Figure S3. Effects of VOR on NK cells. (A) NK92MI cells treated with VOR (0, 1, and 2 μM in blue, purple, and red, respectively) for 24 h were immunolabeled with anti-MICA antibody and fluorescent signals were detected by flow cytometry analysis, with the isotype controls shown as gray histograms. Likewise MICA mRNA level (B) and relative cell viability (C) were measured in NK92MI cells treated with VOR at the indicated concentrations for 24 h.

Supplementary Figure S4. Tumor growth levels *in vivo* over the treatment. The median relative tumor growth levels *in vivo* were demonstrated by box plots at Day 3, 7, 10, 14, 17, 21, and 24.

Supplementary Figure S5. Correlation between metabolism and HDACi-mediated MICA induction. (A) PLC/PRF/5 cells were treated with 2 μM VOR for 48 h and relative *pRB and*

PTEN mRNA levels were quantified by qRT-PCR with normalization to *GAPDH*. (B) PLC/PRF/5 cells were treated with DL-NaL at the indicated concentrations for 5 days and relative *MICA* mRNA levels were quantified by qRT-PCR with normalization to *GAPDH*. (C) PLC/PRF/5 cells were treated with BMS-303141 at the indicated concentrations in the absence or presence of 1.5 μM VOR for 72 h and relative *MICA* mRNA levels were quantified by qRT-PCR with normalization to *GAPDH*.

Supplementary Table

Supplementary Table S1. Transcription factors for MICA expression.

Name	Cell line	Reference
Extracellular signal-regulated kinase	ARK	1
Glycogen synthase kinase 3	Jurkat	2
Heat shock transcription factor 1	HeLa	3
Phosphatidylinositol 3 kinase	PANC-1, BxPC-3	4
Specificity protein 1	HeLa, HepG2	3
E2F*	SKO-007	5
Nuclear factor kappa B*	HeLa	6
STAT3*	SKO-007	7

* Independent of HDACis

Supplementary Table S2. The actual tumor sizes *in vivo*.

Days	Tumor size (mm^2)*			
	Control	Anti-NKG2D	VOR	VOR/ Anti-NKG2D
0	24.06 ± 2.21	18.02 ± 3.69	32.53 ± 16.32	19.46 ± 12.28
3	59.11 ± 11.74	72.17 ± 11.27	56.64 ± 28.34	52.11 ± 27.41
7	80.94 ± 14.84	75.15 ± 18.45	71.12 ± 29.38	62.16 ± 41.72
10	95.76 ± 6.58	92.98 ± 4.37	79.63 ± 32.53	72.85 ± 42.56
14	105.43 ± 10.09	112.14 ± 13.94	83.56 ± 23.42	72.59 ± 40.08
17	111.90 ± 7.73	120.25 ± 10.91	91.15 ± 26.69	79.12 ± 41.27
21	122.19 ± 13.13	130.81 ± 14.68	98.23 ± 28.54	81.18 ± 43.36
24	130.17 ± 13.26	161.51 ± 18.45	116.34 ± 22.11	90.65 ± 44.98

* Means ± SD

Supplementary references

1 Wu, X., Tao, Y., Hou, J., Meng, X. & Shi, J. Valproic acid upregulates NKG2D ligand expression through an ERK-dependent mechanism and potentially enhances NK cell-mediated lysis of myeloma. *Neoplasia* 14, 1178-1189 (2012).

2 Skov, S. *et al.* Cancer cells become susceptible to natural killer cell killing after exposure to histone deacetylase inhibitors due to glycogen synthase kinase-3-dependent expression of MHC class I-related chain A and B. *Cancer Res* 65, 11136-11145 (2005).

3 Zhang, C., Wang, Y., Zhou, Z., Zhang, J. & Tian, Z. Sodium butyrate upregulates expression of NKG2D ligand MICA/B in HeLa and HepG2 cell lines and increases their susceptibility to NK lysis. *Cancer Immunol Immunother* 58, 1275-1285 (2009).

4 Shi, P. *et al.* Valproic acid sensitizes pancreatic cancer cells to natural killer cell-mediated lysis by upregulating MICA and MICB via the PI3K/Akt signaling pathway. *BMC Cancer* 14, 370 (2014).

5 Soriani, A. *et al.* Reactive oxygen species- and DNA damage response-dependent NK cell activating ligand upregulation occurs at transcriptional levels and requires the transcriptional factor E2F1. *J Immunol* 193, 950-960 (2014).

6 Lin, D., Lavender, H., Soilleux, E. J. & O'Callaghan, C. A. NF-kappaB regulates MICA gene transcription in endothelial cell through a genetically inhibitable control site. *J Biol Chem* 287, 4299-4310 (2012).

7 Fionda, C. *et al.* Inhibition of glycogen synthase kinase-3 increases NKG2D ligand MICA expression and sensitivity to NK cell-mediated cytotoxicity in multiple myeloma cells: role of STAT3. *J Immunol* 190, 6662-6672 (2013).
